# Expanding ACMG variant classification guidelines into a general framework

**DOI:** 10.1186/s40246-022-00407-x

**Published:** 2022-08-16

**Authors:** Emmanuelle Masson, Wen-Bin Zou, Emmanuelle Génin, David N. Cooper, Gerald Le Gac, Yann Fichou, Na Pu, Vinciane Rebours, Claude Férec, Zhuan Liao, Jian-Min Chen

**Affiliations:** 1 Univ Brest, Inserm, EFS, UMR 1078, GGB, 22 Avenue Camille Desmoulins, F-29200 Brest, France; 2 Service de Génétique Médicale et de Biologie de la Reproduction, CHRU Brest, F-29200 Brest, France; 3 Department of Gastroenterology, Changhai Hospital, The Secondary Military Medical University, Shanghai, China; 4 Shanghai Institute of Pancreatic Diseases, Shanghai, China; 5 Institute of Medical Genetics, School of Medicine, Cardiff University, Cardiff, UK; 6 Department of Critical Care Medicine, Research Institute of General Surgery, Jinling Hospital, Medical School of Nanjing University, Nanjing, China; 7 Department of Gastroenterology and Pancreatology, Beaujon Hospital, Assistance Publique-Hôpitaux de Paris, Clichy, Université de Paris, Paris, France

**Keywords:** ACMG guidelines, Allele frequency threshold, Allelic heterogeneity, Disease prevalence, Exome sequencing, Genetic heterogeneity, Incomplete penetrance, Multifactorial/complex disease, Pathogenicity, Variant interpretation

## Abstract

**Background:**

The American College of Medical Genetics and Genomics (ACMG)-recommended five variant classification categories (pathogenic, likely pathogenic, uncertain significance, likely benign, and benign) have been widely used in medical genetics. However, these guidelines are fundamentally constrained in practice owing to their focus upon Mendelian disease genes and their dichotomous classification of variants as being either causal or not. Herein, we attempt to expand the ACMG guidelines into a general variant classification framework that takes into account not only the continuum of clinical phenotypes, but also the continuum of the variants’ genetic effects, and the different pathological roles of the implicated genes.

**Main body:**

As a disease model, we employed chronic pancreatitis (CP), which manifests clinically as a spectrum from monogenic to multifactorial. Bearing in mind that any general conceptual proposal should be based upon sound data, we focused our analysis on the four most extensively studied CP genes, *PRSS1*, *CFTR*, *SPINK1* and *CTRC*. Based upon several cross-gene and cross-variant comparisons, we first assigned the different genes to two distinct categories in terms of disease causation: CP-causing (*PRSS1* and *SPINK1*) and CP-predisposing (*CFTR* and *CTRC*). We then employed two new classificatory categories, “predisposing” and “likely predisposing”, to replace ACMG’s “pathogenic” and “likely pathogenic” categories in the context of CP-predisposing genes, thereby classifying all pathologically relevant variants in these genes as “predisposing”. In the case of CP-causing genes, the two new classificatory categories served to extend the five ACMG categories whilst two thresholds (allele frequency and functional) were introduced to discriminate “pathogenic” from “predisposing” variants.

**Conclusion:**

Employing CP as a disease model, we expand ACMG guidelines into a five-category classification system (predisposing, likely predisposing, uncertain significance, likely benign, and benign) and a seven-category classification system (pathogenic, likely pathogenic, predisposing, likely predisposing, uncertain significance, likely benign, and benign) in the context of disease-predisposing and disease-causing genes, respectively. Taken together, the two systems constitute a general variant classification framework that, in principle, should span the entire spectrum of variants in any disease-related gene. The maximal compliance of our five-category and seven-category classification systems with the ACMG guidelines ought to facilitate their practical application.

## Background

Now that the application of exome and genome sequencing in a clinical setting has become fairly routine, we face an increasing challenge in terms of assigning variants to the five discrete classificatory categories (i.e., “pathogenic”, “likely pathogenic”, “uncertain significance”, “likely benign”, and “benign”) [1] recommended by the American College of Medical Genetics and Genomics and the Association for Molecular Pathology (ACMG-AMP; referred to henceforth as ACMG). A fundamental issue is that the ACMG guidelines were specifically drawn up in order to describe variants identified in genes underlying Mendelian disorders. However, in reality, the etiology of a given disorder may (1) lie on a spectrum from highly penetrant single gene defect to multifactorial disease and (2) involve multiple gene loci that do not make comparable pathological contributions to the disease in question. Moreover, even in genes underlying Mendelian disorders, clinically relevant variants do not readily fall into a discontinuous causal versus benign dichotomy [2]. Indeed, as opined by Wright and colleagues [3], some basic conceptual questions about variant interpretation still remain to be addressed in medical genetics. Thus, should the term “pathogenic” be generally applied to any disease-relevant variant in a given disease-causing gene? When should a pathologically relevant mutation be considered to be a “risk” variant rather than being “pathogenic” in its own right? Various adaptations and refinements of the ACMG guidelines have previously been made in the context of secondary findings derived from clinical exome and genome sequencing [4] as well as in the context of different genes/diseases [5–14] or specific variant types [15]. In addition, a comprehensive refinement of the ACMG variant classification criteria in terms of 40,000 clinically observed variants has also been made [16]. However, in our view, none of these provide a general framework that adequately addresses the aforementioned conceptual issues. Very recently, an “ABC system” (involving both functional and clinical grading steps) has been proposed for the classification of all types of genetic variant (including hypomorphic alleles, imprinted alleles, copy number variants, runs of homozygosity, enhancer variants and variants related to traits) [17]. However, a key limitation of this system is that it relies upon quite different codes (i.e., A, B, C,…) for variant classificatory categories from those used by ACMG, which will likely hamper cross-comparison and may well lead to widespread confusion.

**Fig. 1:**
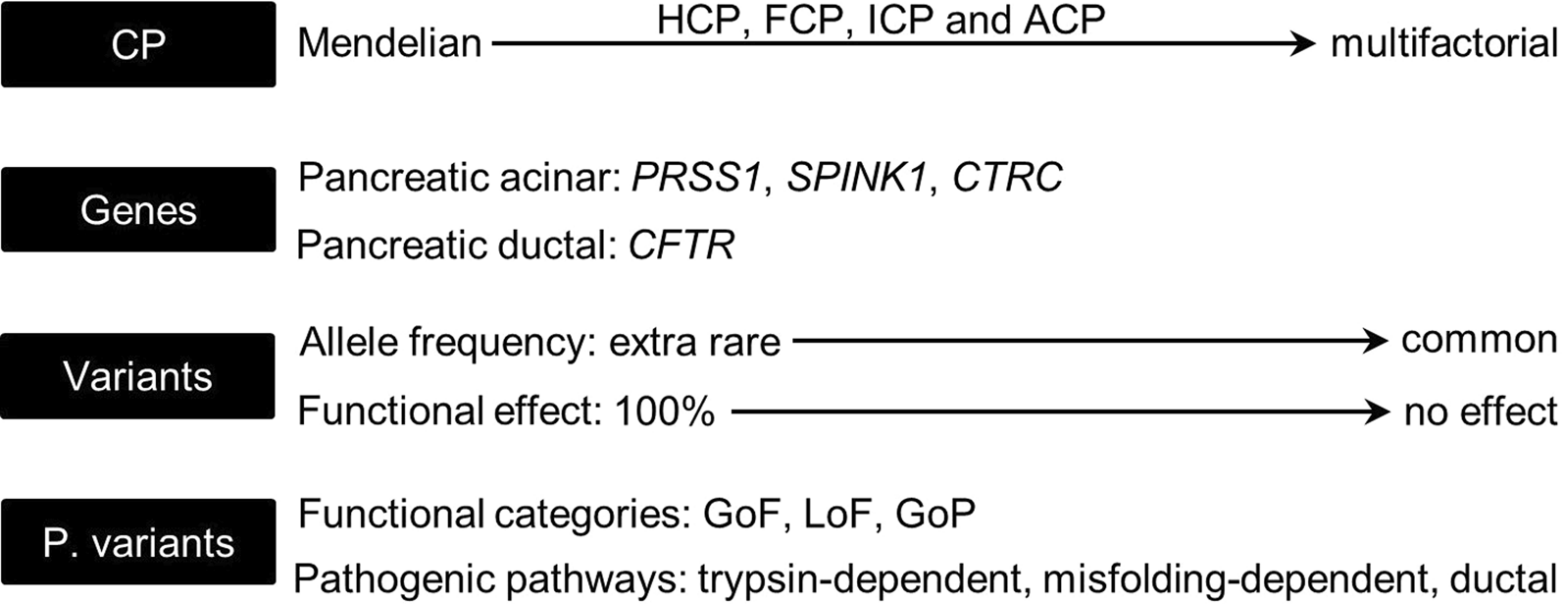
Layers of complexity challenging variant classification in CP that were included for analysis in the current study. *CP* chronic pancreatitis, *HCP* hereditary CP, *FCP* familial CP, *ICP* idiopathic CP, *ACP* alcoholic CP, *P* variants, pathological variants, *GoF* gain-of-function, *LoF* loss-of-function, *GoP* gain-of-proteotoxicity

Herein, we propose a general variant classification framework that takes into account the continuum of clinical phenotypes, the continuum of the variants’ genetic effects, and the different pathological roles of the implicated genes, while maximally complying with ACMG guidelines. To this end, we opted to employ chronic pancreatitis (CP) as a disease model. CP, a chronic inflammatory process of the pancreas that leads to irreversible morphological changes and the progressive impairment of both exocrine and endocrine functions, can be caused by both genetic and environmental factors [18, 19]. In common with many other diseases, the process of genetic discovery in CP began with the mapping and identification of a causative gene (i.e., *PRSS1* (OMIM #276000; encoding cationic trypsinogen)) for a Mendelian form of the disease, autosomal dominant hereditary pancreatitis [20–23]. Thereafter, a diverse range of variants in more than 10 different genes (for references, see Masson et al. [24]) have been identified in patients with hereditary, familial, idiopathic and/or alcoholic CP (see Main text for disease subtype definitions). These different forms of CP may be considered to reflect a continuum of the disease extending from monogenic to multifactorial [25], thereby rendering CP an archetypal model of a genetic disease (Fig. 1).

A preprint of this manuscript has been posted on medRxiv [26].

## Main text

### Genes included in the analysis

A general conceptual proposal should be based upon sound data. We therefore opted to focus our analysis on the first four discovered and most extensively studied CP genes (i.e., *PRSS1*, *CFTR* (OMIM #602421; encoding cystic fibrosis transmembrane conductance regulator), *SPINK1* (OMIM #167790; encoding pancreatic secretory trypsin inhibitor) and *CTRC* (OMIM #601405; encoding chymotrypsin C)), each of which is known to harbor a large number of pathologically relevant variants [23, 27–37]. General information about these four genes, including year and method of gene discovery, mRNA reference accession number, length of coding DNA sequence and length of the encoded protein, may be found in Table 1.

**Table 1:** Some general information about the four CP genes

Gene (encoded protein)	Year of discovery	Discovery approach	Reference mRNA sequence	Coding sequence (bp)	Protein sequence (aa)	Protein tissue expression^a^	Cell type expression in exocrine pancreas	Functional categories of pathologically relevant variants^b^	o/e score of pLoF variants (95% CI)^c^
*PRSS1* (cationic trypsinogen)	1996	Positional cloning [23]	NM_002769.5	744	247	Specifically expressed in exocrine pancreas	Acinar	GoF (majority); GoP (minority)	1.31 (0.86–1.86)
*CFTR* (cystic fibrosis transmembrane conductance regulator)	1998	Candidate gene approach based upon the role of *CFTR* in cystic fibrosis [28, 29]	NM_000492.4	4443	1480	Highly expressed in exocrine pancreas and kidney; medially expressed in salivary gland, duodenum and small intestine	Ductal and centroacinar	LoF	1.09 (0.91–1.31)
*SPINK1* (pancreatic secretory trypsin inhibitor)	2000	Candidate gene approach stimulated by the *PRSS1* finding [30]	NM_001379610.1	240	79	Highly expressed in exocrine pancreas, gastrointestinal tract, urinary bladder and appendix	Acinar	LoF	0.24 (0.09–1.13)
*CTRC* (chymotrypsin C)	2008	Candidate gene approach stimulated by the *PRSS1* finding [31, 32]	NM_007272.3	807	268	Specifically expressed in exocrine pancreas	Acinar	LoF (majority); GoP (minority)	1.15 (0.78–1.69)

*aa* amino acid, *bp* base-pair, *CI* confidence interval, *CP* chronic pancreatitis, *GoF* gain-of-function, *GoP* gain-of-proteotoxicity, *LoF* loss-of-function, *o/e* observed/expected, *pLoF* predicted loss-of-function

^a^In accordance with the Human Protein Atlas (https://www.proteinatlas.org/) [73]

^b^See text for details

^c^In accordance with gnomAD v2.1.1 (https://gnomad.broadinstitute.org/) [74]

### Variants in the four CP genes considered here

For reported variants in the *PRSS1*, *SPINK1* and *CTRC* genes, the reader is referred to the Genetic Risk Factors in Chronic Pancreatitis Database [38]. CP-associated variants in the *CFTR* genes were sought in PubMed using a keyword search (i.e., *CFTR* plus pancreatitis plus variant or *CFTR* plus pancreatitis plus mutation; the latest search was performed on 12 April 2022). Data from some original reports were reinterpreted in accordance with the disease subtype definitions outlined below.

### Disease subtype definitions

CP cases empirically demonstrated to have a genetic contribution may be classified into four distinct subtypes, namely hereditary CP (HCP), familial CP (FCP), idiopathic CP (ICP) and alcoholic CP (ACP). The first three subtypes were defined in accordance with our previous practice. Specifically, HCP is defined in terms of having three or more affected family members spanning at least two generations, whereas FCP is indicated by a positive family history without satisfying the strict diagnostic criteria for HCP; ICP is indicated when neither a positive family history of pancreatitis nor any obvious external causative risk factors (e.g., excessive alcohol consumption, infection, trauma or drug use) have been reported [25, 27, 39]. ACP was defined in accordance with the original publications, in which it was usually attributed to an alcohol intake of ≥ 80 g/d for a male and ≥ 60 g/d for a female for at least 2 years. “Non-alcoholic CP”, a term used in some publications, may be regarded as being equivalent to ICP, and indeed this has been our previous practice [40]. Finally, it should be emphasized that ICP was defined in terms of the absence of any identifiable etiology prior to genetic analysis.

### Classifying the pathologically relevant variants in the four CP genes into three categories in terms of their functional consequences

**Fig. 2:**
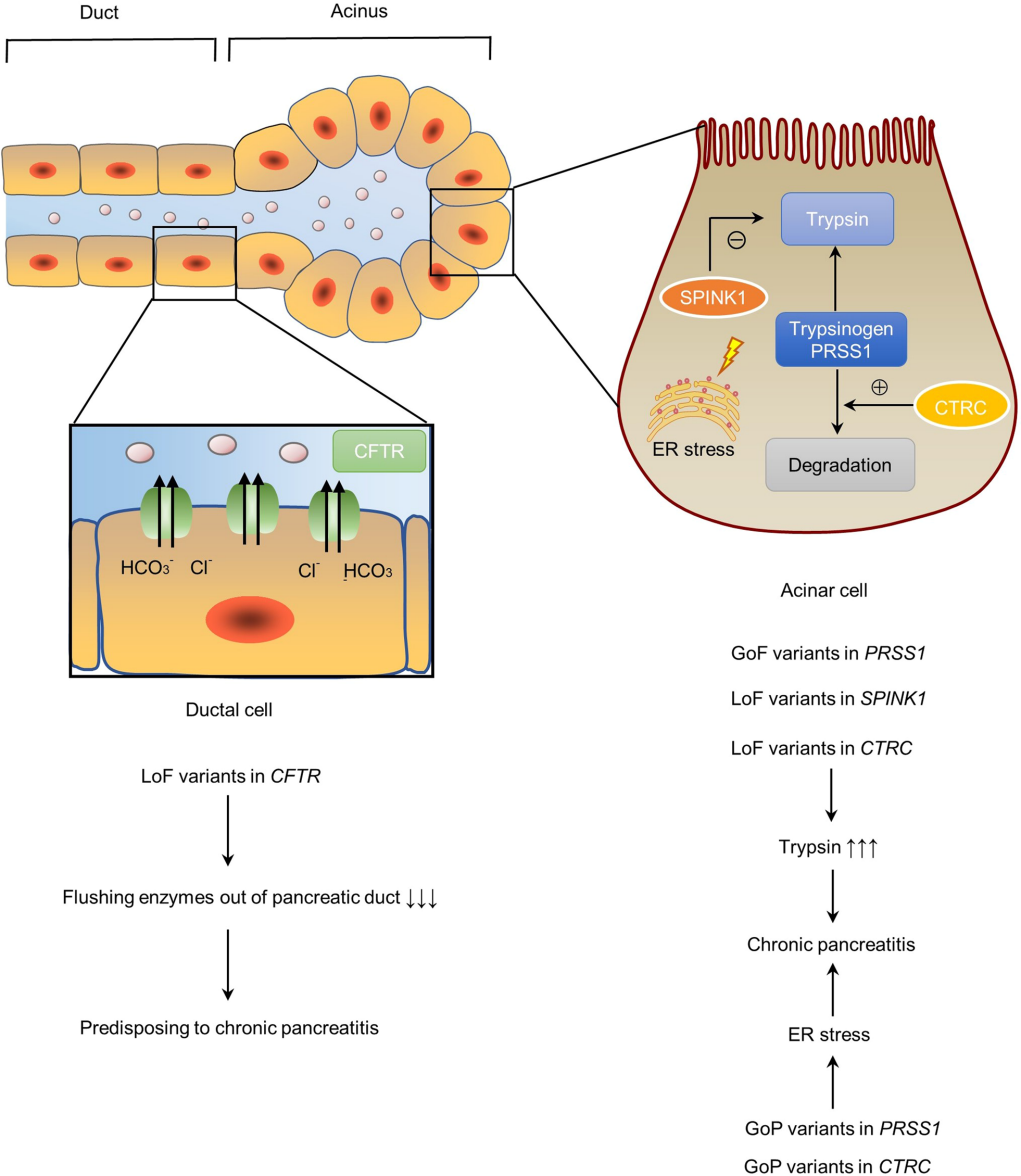
Illustration of the cellular locations of PRSS1, CFTR, CTRC and SPINK1 within the exocrine pancreas and the pathological mechanisms underlying the chronic pancreatitis-related variants in the corresponding genes. *ER* endoplasmic reticulum, *GoF* gain-of-function, *LoF* loss-of-function, *GoP* gain-of-proteotoxicity

*PRSS1*, *SPINK1* and *CTRC* are specifically or highly expressed in the acinar cells, whereas *CFTR* is highly expressed in the ductal cells of the exocrine pancreas (Table 1; Fig. 2). Based upon current knowledge, all pathologically relevant variants in the four CP genes may be classified into three functional categories: gain-of-function (GoF), loss-of-function (LoF) and gain-of-proteotoxicity (GoP). Briefly, GoF variants in *PRSS1* result in increased trypsinogen activation and/or increased trypsin stability. These variants, as well as LoF variants in *SPINK1* and *CTRC* (NB. SPINK1 specifically inhibits trypsin, whereas CTRC specifically degrades trypsinogen/trypsin), give rise to increased intrapancreatic trypsin activity or a gain of *trypsin* within the pancreas, thereby causing or predisposing to CP (trypsin-dependent pathway) [39, 41]. A small subset of pathologically relevant variants in *PRSS1* and *CTRC* induced the misfolding of their corresponding zymogens and elicited endoplasmic reticulum (ER) stress in the pancreatic acini (misfolding-dependent pathway) [42]; these variants are termed GoP. In the exocrine pancreas, CFTR regulates cAMP-mediated bicarbonate secretion into the pancreatic duct lumen, which dilutes and alkalinizes the protein-rich acinar secretions; LoF variants in CFTR are thought to lead to slowed flushing of trypsinogen/trypsin out of the pancreatic ducts, thereby predisposing to pancreatic injury and CP [25] (termed “ductal pathway” by Mayerle and colleagues [43]). These classifications served as the basis to perform the cross-gene and cross-variant comparisons outlined below.

### Classifying the four CP genes into two distinct categories in terms of causation

The four CP genes do not contribute equally to the pathophysiology of the exocrine pancreas. To distinguish their roles in the pathogenesis of CP at the gene level, we firstly sought to determine whether the very rare variants [defined as having a minor allele frequency (MAF) of < 0.001 in accordance with Manolio et al. [44] in any gnomAD (Genome Aggregation Database) subpopulation)] were identified in the Mendelian form of CP or HCP in the context of each gene. A MAF cutoff of 0.001 has previously been recommended for filtering variants responsible for dominant Mendelian disorders [45]. The MAF of < 0.001 corresponds to a carrier frequency of < 0.002. It was used here as a very conservative cutoff given that it was more than 600 times higher than the prevalence of HCP, which was estimated to be 0.3/100 000 in Western Countries [46]. The premise was that such variants, where presumed (or experimentally demonstrated) to fall into the aforementioned GoF, GoP or LoF categories, can be confidently interpreted as disease-causing.

*PRSS1* was the first CP gene to be identified, with multiple very rare variants including GoF copy number and missense variants and GoP missense variants (*n* = 12; Table 2) subsequently being reported in many HCP families. By contrast, only a limited number of very rare *SPINK1* variants (*n* = 3; Table 2), and not particularly very rare *CFTR* and *CTRC* variants (*n* = 0; Table 2), have been reported in HCP families. Moreover, the HCP families harboring *PRSS1* mutations were generally large, often involving ≥ 4 patients across ≥ 3 generations, whereas the HCP families harboring *SPINK1* mutations had at most 3 patients over 3 generations (Table 2). In short, high-confidence disease-causing variants were found in *PRSS1* and *SPINK1* but not in *CFTR* and *CTRC*.

**Table 2:** Very rare pathologically relevant variants found in HCP in the context of four CP genes

Gene	Variant^a^	Number of HCP families (family description) reported^b^	Reference(s)	Biological/functional consequence	gpAF (hspAF) in gnomAD^c^
*PRSS1*	Trypsinogen gene triplication	5 (10 patients across 4 generations^d^)	Le Maréchal et al. [27]	GoF (gene dosage) [39]	Absent
	Double “gain-of-function” hybrid variant	1 (6 patients across 3 generations)	Masson et al. [75]	GoF (gene dosage plus effect of p.Asn29Ile)	Absent
	c.47C > T (p.Ala16Val)	2 (4 patients across 2 generations; 3 patients across 2 generations)	Grocock et al. [76]	GoF (increased activation) [77]	Absent [78]
	c.62A > C (p.Asp21Ala)	1 (5 patients across 3 generations)	Yilmaz et al. [79]	GoF (increased activation) [80]	Absent
	c.63_71dup (p.Lys23_Ile24insIleAspLys	1 (3 patients across 2 generations)	Joergensen et al. [81]	GoF (increased activation) [81]	Absent
	c.86A > T (p.Asn29Ile)	The second most frequent variant causing HCP [38]; in the first report, one family had 19 patients across 7 generations [82]	Gorry et al. [82]	GoF (increased activation and stability) [77]	Absent
	c.86A > C (p.Asn29Thr)	1 (8 patients across 3 generations)	Dytz et al. [83]	GoF (increased activation and stability) [77]	Absent
	c.116T > C (p.Val39Ala)	1 (9 patients across 3 generations)	Arduino et al. [84]	GoF (increased stability) [77]	Absent
	c.311T > C (p.Leu104Pro)	2 (both having 3 patients across 3 generations)	Teich et al. [85]; Németh et al. [86]	GoP (intracellular retention and elevation of ER stress marker) [87]	Absent
	c.346C > T (p.Arg116Cys)	2 (3 patients across 2 generations; 3 patients across 3 generations)	Pho-Iam et al. [88]; Kereszturi et al. [89]	GoP (intracellular retention and elevation of ER stress marker) [89]	0.00007072 (0.0007018, East Asian)
	c.365G > A (p.Arg122His)	The most frequent variant causing HCP [38]; in the discovery report, one family had 20 patients across 4 generations [23]		GoF (increased activity) [90, 91]	0.00001194 (0.00002639, non-Finnish European)
	c.365_366GC > AT (p.Arg122His)	1 (4 patients across 4 generations)	Howes et al. [92]	Same as above	Absent
*CFTR*	Not identified				
*SPINK1*	c.27DelC (p.Ser10ValfsTer5)	1 (3 patients across 2 generations)	Le Maréchal et al. [93]	LoF (predicted complete functional loss)	0.00001197 (0.00002896, Latino/Admixed American)
	c.41T > G (p.Leu14Arg)	2 (both having 3 patients across 3 generations)	Király et al. [94]	LoF (experimentally demonstrated to abolish SPINK1 secretion) [94]	Absent
	Deletion of the entire gene	1 (3 patients across 2 generations)	Masson et al. [95]	LoF (predicted complete functional loss)	Absent
*CTRC*	Not identified				

*CP* chronic pancreatitis, *gpAF* global population allele frequency, *GoF* gain-of-function, *GoP* gain-of-proteotoxicity, *HCP* hereditary CP, *hspAF* highest subpopulation allele frequency, *LoF* loss-of-function

^a^All are heterozygous. See Table 1 for reference mRNA accession numbers

^b^Data from some original reports were reinterpreted in accordance with our working definition of HCP

^c^In accordance with gnomAD v2.1.1 or SVs v2.1 (https://gnomad.broadinstitute.org/) [74]

^d^Described was the family with the most affected patients

The abovementioned findings may have been influenced by many factors including differences in patient recruitment and mutation analysis protocols between laboratories and different timespans since the first report of CP gene discovery. To confirm or refute these findings, we performed three additional comparative analyses. Firstly, we compared the observed/expected (*o*/*e*) scores of predicted LoF (pLoF) variants in the four genes from gnomAD v2.1.1 (Table 1). The *o*/*e* score is an indicator of LoF intolerance devised by Karczewski and colleagues [47], low *o*/*e* values being indicative of strong intolerance. The highest *o*/*e* score was exhibited by *PRSS1* (*o*/*e* = 1.31); this is understandable because it is predominantly GoF variants in this gene that cause CP, whereas LoF variants in *PRSS1* and *PRSS2* (encoding anionic trypsinogen, the second major isoform after cationic trypsinogen) are protective with respect to CP [48, 49]. With regard to the latter, we evaluated the pLoF *PRSS1* variants in gnomAD v2.1.1. The highest subpopulation allele frequency (hspAF) of such variants, which was found in the case of the c.200 + 1G > A variant, was 0.02871 (African/African American). In the context of the three genes for which LoF variants (or predominantly LoF variants) underlie the disease*, CFTR* and *CTRC* have an o/e score of > 1 (1.09 and 1.15, respectively), whereas *SPINK1* has an o/e score of < 1 (specifically, 0.24).

Secondly, we compared the odds ratios (ORs) calculated from the aggregated pathologically relevant variants in the three genes for which LoF variants (or predominantly LoF variants) underlie the disease. For reasons of simplicity and comparability, we used data from a German study that analyzed these genes in a large cohort of patients (*n* = 410–660) and controls (*n* = 750–1758) [34]. The ORs for *CFTR*, *CTRC* and *SPINK1* variants were 2.7, 5.3 and 15.6, respectively. In other words, the aggregated pathologically relevant variants in the *CFTR* and *CTRC* genes were associated with a much lower genetic effect than those in the *SPINK1* gene.

Thirdly, and reinforcing the above point, even the most severe LoF variants in *CFTR* and *CTRC* do not exert a very large genetic effect. Thus, for example, *CFTR* p.Phe508del, the classical cystic fibrosis-causing variant, had an OR of only 2.5 (95% CI 1.7–3.9) for CP [34]. In similar vein, *CTRC* p.Lys247_Arg254del, which results in a complete loss of CTRC enzymatic activity, had an OR of 6.4 (95% CI 2.3–17.5) [50]. By contrast, the OR for ICP conferred by *SPINK1* c.194 + 2T > C, which should result in a ~ 90% functional loss of SPINK1 activity [51, 52], was 59.31 (95% CI 33.93–103.64) based upon data from a Chinese study [36, 40].

Finally, a remarkable difference in terms of phenotype expression was observed between naturally occurring human *SPINK1* and *CTRC* knockouts. Two *SPINK1* knockouts, one a homozygous deletion of the entire *SPINK1* gene, the other the homozygous insertion of a full-length inverted *Alu* element into the 3′-untranslated region of the *SPINK1* gene (experimentally determined to cause the complete loss of *SPINK1* expression), presented with severe exocrine pancreatic insufficiency around 5 months of age [53]. By contrast, a *CTRC* knockout, homozygous for a deletion of the entire *CTRC* locus, had been clinically asymptomatic until adulthood [54]. Only at the age of 20 was he incidentally found to have calcifications and cysts in the pancreas; subsequent laboratory tests revealed exocrine pancreatic insufficiency [54]. These highly unusual cases are strongly consistent with SPINK1 exerting a much stronger effect than CTRC in terms of the negative regulation of the level of prematurely activated trypsin within the pancreas.

**Fig. 3:**
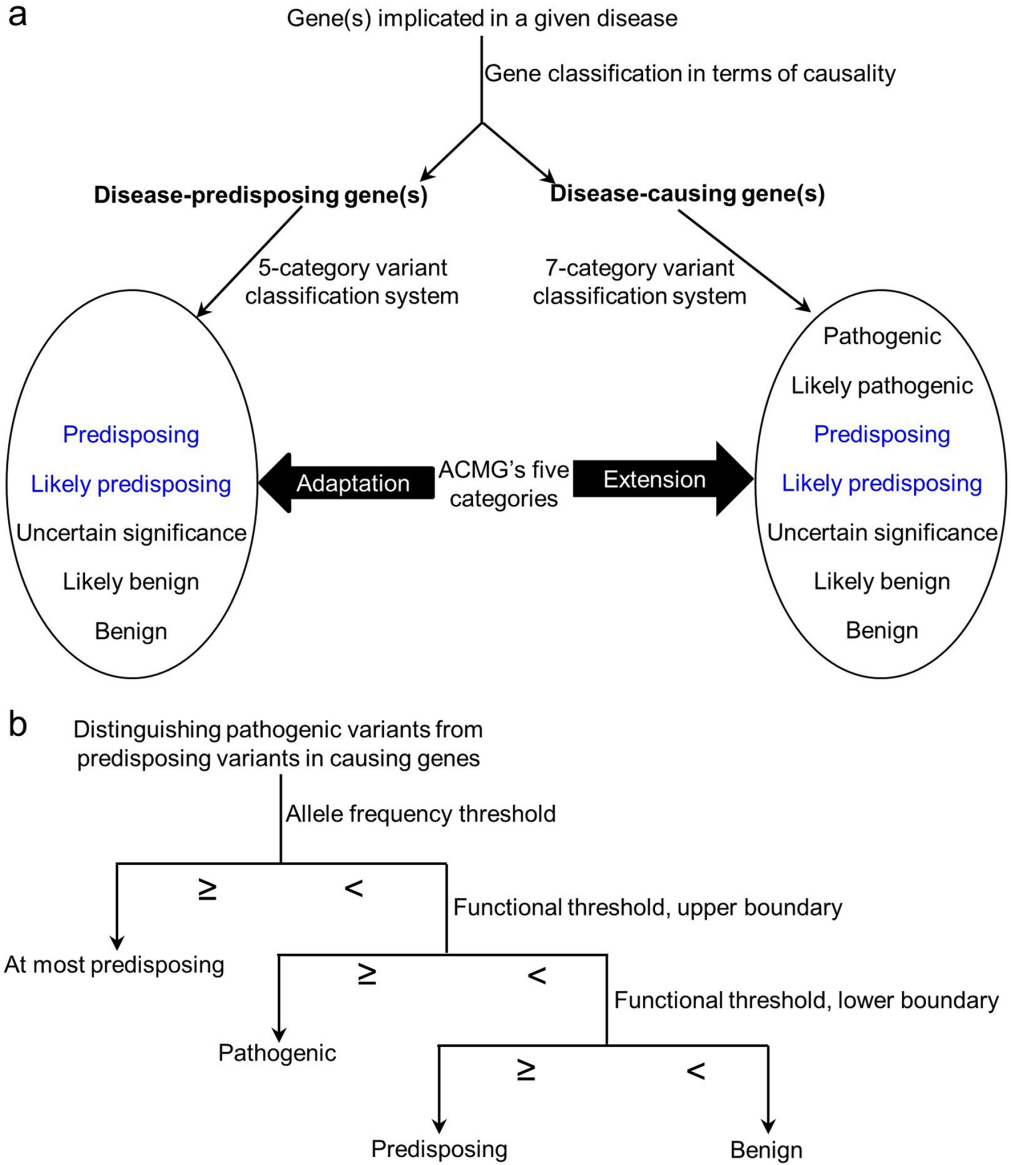
Key components of our proposed general variant classification framework. **a** Disease genes were first classified into either “causing” or “predisposing” based upon multiple sources of evidence. Then, minimal extension and adaptation were made to the five ACMG variant classificatory categories in the different gene contexts. The two new categories proposed in this study are highlighted in blue. **b** Illustration of the use of two thresholds to distinguish pathogenic variants from predisposing variants in disease-causing genes

Taking these observations together, we classified *PRSS1* and *SPINK1* as CP-causing genes and *CFTR* and *CTRC* as CP-predisposing genes. This step, generalized to any gene(s) implicated in any disease, is illustrated in Fig. 3a.

### Adapting ACMG guidelines for the classification of variants in the two CP-predisposing genes

We would propose to change two of the five ACMG categories, “pathogenic” and “likely pathogenic”, to “predisposing” and “likely predisposing” for the purposes of classifying the pathologically relevant variants in the *CFTR* and *CTRC* genes. Thus, all *CFTR* pathologically relevant variants previously known as “cystic fibrosis-causing, severe”, “cystic fibrosis-causing, mild” and “non-cystic fibrosis-causing” [34] will be classified as “predisposing” in the context of CP, with the conventional cystic fibrosis-based categories being provided in parentheses. As for the *CTRC* variants, we propose to reclassify all “pathogenic” variants listed in the Genetic Risk Factors in Chronic Pancreatitis Database [38] as CP “predisposing”.

A generalized five-category classification system in terms of disease-predisposing genes is illustrated in Fig. 3a.

### Extending the ACMG guidelines to classify variants in the two CP-causing genes

It is evident that not all pathologically relevant variants in a given disease-causing gene are causative. To make a distinction at this juncture, we propose to add the above-mentioned two novel categories, “predisposing” and “likely predisposing”, to the five ACMG categories (Fig. 3a). Therefore, the key issue is how to distinguish “pathogenic” from “disease predisposing” among the pathologically relevant variants in the causative genes (Fig. 3b).

#### Establishing an allele frequency threshold to distinguish pathogenic variants from disease predisposing variants

The relative rarity of a variant is a proxy indicator of its potential pathogenicity [1, 55–59]. But defining an allele frequency threshold above which a pathological variant should be considered too common to cause the disease in question is inherently challenging owing to the uncertainties pertaining to disease prevalence, the variable mode of inheritance, the existence of genetic and allelic heterogeneity, and the issue of incomplete penetrance [58].

Earlier, we used a conservative MAF cutoff of < 0.001 to evaluate high confidence HCP-causing variants. Herein, we further explore this issue by evaluating the population allele frequencies of what we term “gold-standard” pathologically relevant variants in the two CP-causing genes. “Gold-standard” LoF variants in *SPINK1* refer to pLoF variants or variants experimentally shown to result in a complete or almost complete (> 95%) loss of SPINK1 function. By contrast, it is impractical to quantify the effect of GoF or GoP variants. Keeping this *caveat* in mind, “gold-standard” GoF variants in *PRSS1* refer to those variants that are very rare and which have been experimentally shown to increase trypsinogen activation and/or trypsin stability, whereas “gold-standard” GoP variants in *PRSS1* refer to those variants that are very rare and which have experimentally been shown to reduce protein secretion and elicit ER stress. The global population allele frequency (gpAF) and hspAF of these “gold-standard” variants are provided in Tables 3, 4 and 5. Herein, it should be noted that in the context of “gold-standard” LoF variants in *SPINK1* (Table 5), p.Arg67His, which was experimentally shown to cause a complete functional loss of SPINK1 [60], has a hspAF as high as 0.03078. This apparent outlier was excluded from the final analysis.

**Table 3:** “Gold-standard” GoF variants in *PRSS1*

Variant		gpAF in gnomAD^a^	hspAF in gnomAD^a^
Nucleotide change	Amino acid change		
Triplication CNV		Absent	
Duplication CNV		Absent	
Double “gain-of-function” CNV		Absent	
c.47C > T	p.Ala16Val	Absent [78]	
c.49C > A	p.Pro17Thr	Absent	
c.56A > C	p.Asp19Ala	Absent	
c.62A > C	p.Asp21Ala	Absent	
c.65A > G	p.Asp22Gly	Absent	
c.68A > G	p.Lys23Arg	Absent	
c.63_71dup	p.Lys23_Ile24insIleAspLys	Absent	
c.86A > T	p.Asn29Ile	Absent	
*PRSS1-PRSS2* hybrid (gene conversion)	p.Asn29Ile + p.Asn54Ser	Absent	
c.86A > C	p.Asn29Thr	Absent	
*PRSS1-PRSS2* hybrid (gene conversion)	p.Asn29Ile + p.Asn54Ser	Absent	
c.116 T > C	p.Val39Ala	Absent	
c.276G > T	p.Lys92Asn	0.000007953	0.00006152 (African/African American)
c.364C > T	p.Arg122Cys	0.00001988	0.00003517 (non-Finnish European)
c.365G > A	p.Arg122His	0.00001194	0.00002639 (non-Finnish European)
c.365_366GC > AT	p.Arg122His	Absent	

See the Genetic Risk Factors in Chronic Pancreatitis Database [38] for original genetic and functional analysis reports

*GoF* gain-of-function, *gpAF* global population allele frequency, *hspAF* highest subpopulation allele frequency

^a^In accordance with gnomAD v2.1.1 or SVs v2.1 (https://gnomad.broadinstitute.org/) [74]

**Table 4:** “Gold-standard” GoP variants in *PRSS1*

Variant		gpAF in gnomAD^a^	hspAF in gnomAD^a^
Nucleotide change	Amino acid change		
c.311T > C	p.Leu104Pro	Absent	
c.346C > T	p.Arg116Cys	0.00007072	0.0007018 (East Asian)
c.415T > A	p.Cys139Ser	Absent	
c.416G > T	p.Cys139Phe	Absent	

See the Genetic Risk Factors in Chronic Pancreatitis Database [38] for original genetic and functional analysis reports

*GoP* gain-of-proteotoxicity, *gpAF* global population allele frequency, *hspAF* highest subpopulation allele frequency

^a^In accordance with gnomAD v2.1.1 or SVs v2.1 (https://gnomad.broadinstitute.org/) [74]

**Table 5:** “Gold-standard” LoF variants in *SPINK1*

Variant		gpAF in gnomAD^a^	hspAF in gnomAD^a^
Nucleotide change	Amino acid change		
*Presumed complete functional loss*			
c.-28,211_*2,066del		Absent	
c.-15,969_*7,702del		Absent	
c.-320_c.55 + 961del		Absent	
c.2 T > G	p.Met1?	Absent	
c.2 T > C	p.Met1?	Absent	
c.27delC	p.Ser10ValfsTer5	0.00001197	0.00002896 (Latino/Admixed American)
c.55 + 1G > A		Absent	
c.87 + 1G > A		Absent	
c.98_99insA	p.Tyr33Ter	Absent	
c.177delG	p.Val60TyrfsTer35	Absent	
c.194 + 1G > A		Absent	
*Experimentally demonstrated complete or almost complete functional loss*			
c.41T > C	p.Leu14Pro	Absent	
c.41T > G	p.Leu14Arg	Absent	
c.123G > C	p.Lys41Asn^b^	Absent	
c.143G > A	p.Gly48Glu	Absent	
c.150T > G	p.Asp50Glu	0.000003991	0.000008834 (non-Finnish European)
c.160T > C	p.Tyr54His	Absent	
c.190A > G	p.Asn64Asp	Absent	
c.198A > C	p.Lys66Asn	0.0002272	0.0004129 (non-Finnish European)
c.199C > T	p.Arg67Cys	Absent	
c.200G > A	p.Arg67His^c^	0.003187	0.03078 (African/African American)
c.206C > T	p.Thr69Ile	0.00001198	0.0001635 (East Asian)
c.236G > T	p.Cys79Phe	Absent	
c.*14_c.*15ins359		Absent	

See the Genetic Risk Factors in Chronic Pancreatitis Database [38] for original genetic and functional analysis reports

*gpAF* global population allele frequency, *hspAF* highest subpopulation allele frequency, *LoF* loss-of-function

^a^In accordance with gnomAD v2.1.1 or SVs v2.1 (https://gnomad.broadinstitute.org/) [74]

^b^Functional analysis of this variant was performed in ref. [66]

^c^This variant was regarded as an outlier and was therefore excluded from the final analysis

As shown in Tables 3, 4 and 5, only a small subset (precisely 19% (9/47)) of the “gold-standard” pathologically relevant variants in the two CP-causing genes were found in normal populations. Of this small set of variants, the high-confidence HCP-causing *PRSS1* p.Arg116Cys has the highest hspAF (0.0007018). We therefore elected to adopt the previously recommended allele frequency of 0.001 for the filtering of dominant Mendelian disorders [45] as the threshold hspAF for differentiating pathogenic from disease predisposing variants in the *PRSS1* and *SPINK1* genes.

#### Establishing gene-specific functional thresholds to distinguish pathogenic variants from disease predisposing variants

In the two CP-causing genes, not all pathologically relevant variants with a hspAF of < 0.001 can be pathogenic due to their different functional effects. Taking into consideration the different roles of the two genes, we attempted to set gene-specific functional thresholds that would allow pathogenic variants to be distinguished from disease predisposing variants.

As mentioned earlier, it is impractical to quantify the functional effect of GoF or GoP variants in the *PRSS1* gene. Given (1) the central role of PRSS1 in the trypsin-dependent pathway and (2) that *PRSS1* is the most abundantly expressed of the pancreatic zymogen genes, we would tentatively classify all *PRSS1* variants with an allele frequency of < 0.001 that have been experimentally demonstrated to be consistent with a GoF or GOP mechanism, as pathogenic.

We would further propose that those *SPINK1* variants with an allele frequency of < 0.001, that were either presumed or experimentally shown to cause a complete or almost complete functional loss (> 95%) of SPINK1, should be regarded as pathogenic. Additional support for this proposal came from the *SPINK1* c.194 + 2T > C variant which is associated with a ~ 90% functional loss of SPINK1 [51, 52] but has an hspAF of 0.003335 in the East Asian population. As for the lower boundary of functional loss for defining disease predisposing *SPINK1* variants, we would tentatively propose a functional loss of at least 10%.

#### Use of the two newly established thresholds to reclassify several variants in the two CP-causative genes

In the Genetic Risk Factors in Chronic Pancreatitis Database [38], variants in the *PRSS1* and *SPINK1* genes are systematically classified in accordance with the ACMG recommended five categories with the addition of a new “protective” category. Herein, we mainly focus on the missense variants and pLoF variants that were classified as “pathogenic” or “likely pathogenic” in *PRSS1* and *SPINK1* by the Database [38]. Utilizing the newly established thresholds would result in the reclassification of multiple variants, as described below.

In the context of *PRSS1*, p.Gly208Ala would be reclassified from “pathogenic” to “disease predisposing”, primarily because its hspAF is 0.00987 (East Asian), ~ 10 times higher than the 0.001 allele frequency threshold; moreover, functional assays revealed that this variant had only a moderate impact on secretion [61]; finally, in terms of its genetic effect, it had an OR of only 4.92 for ICP [36, 40]. The “pathogenic” p.Lys92Asn and p.Ser124Ser variants would be reclassified as “likely pathogenic” since both showed moderate impact on secretion but no data on ER stress were available. We would also propose to reclassify the “protective” LoF variants p.Tyr37Ter and c.200 + 1G > A as “benign”, with a view to avoiding the addition of a clinically irrelevant category to the five pre-existing ACMG categories. Nevertheless, to distinguish them from the classical “benign” variants (e.g., missense variants that have been experimentally demonstrated to be functionally neutral), the “protective” nature of these LoF variants in *PRSS1* may be specified in parentheses after the “benign” category (Table 6). Employing the same line of reasoning, we would propose to use the risk allele rather than the protective allele for variant classification with respect to the common promoter variant located at c.-204, upstream of the translational initiation codon of *PRSS1* [62–64]. Consequently, c.-204C > A (protective) should be described as c.-204A > C (predisposing).

**Table 6:** Illustrative examples of additions to the main classification categories in the context of *PRSS1* variants

Variant	Classification
Trypsinogen gene triplication	Pathogenic (causes HCP; has also been noted in cases with FCP and ICP; causes the disease via a gene dosage effect) [39]
p.Ala16Val	Pathogenic (highly variable penetrance [38]; causes disease via the trypsin-dependent pathway) [77]
p.Arg122His	Pathogenic (the most frequent variant found in HCP families [38]; causes disease via the trypsin-dependent pathway) [41, 91]
p.Gly208Ala	Predisposing (Asian population-specific variant, with an allele frequency of 0.009873 in East Asians; odds ratio for ICP, 4.92 [36]; may predispose to CP through the misfolding pathway [42] since it causes a moderate effect on secretion [61])
c.-204A > C	Predisposing (a common promoter polymorphism whose pathological authenticity is supported by both in silico and functional data; exerts a moderate genetic effect; odds ratio for ICP, 1.28) [64]
c.200 + 1G > A	Benign (a loss-of-function mutation that was found in normal controls; protective against CP)

*CP* chronic pancreatitis, *FCP* familial CP, *GoF* gain-of-function, *HCP* hereditary CP, *ICP* idiopathic CP

In the context of *SPINK1* variants, there would be three noteworthy reclassifications. First, the abovementioned c.194 + 2T > C should be reclassified from “pathogenic” to “predisposing”. Second, the extensively studied p.Asn34Ser variant should be reclassified from “likely benign” to “benign’ [65–67]. Third, the functional enhancer variant, c.-4141G > T, which is in extensive linkage disequilibrium with p.Asn34Ser [65, 67], should be reclassified from “likely pathogenic” to “predisposing” owing to its hspAF of ~ 0.01975 (South Asia). Additionally, a very rare *SPINK1* variant, p.Arg65Gln, which has been shown to cause a ~ 50% functional loss of SPINK1 [68, 69], would be also reclassified from “pathogenic” to “predisposing” based upon the above established *SPINK1*-specific functional threshold (functional loss of > 10 to < 95%).

### Further additions to the general classification framework

As mentioned above, it is desirable to provide necessary information (such as detection frequency in patients, reported OR, functional analytic data, etc.) about the pathologically relevant variant in question in parentheses immediately after the variant’s principal classification. The main reason is that, for any given disease gene, there are often a large number of variants classified as either “pathogenic” or “predisposing”. We provide illustrative examples in the context of *PRSS1* variants in Table 6.

## Discussion

Employing CP as a disease model and focusing on the four firmly established CP genes, we propose a general variant classification framework that both complements and extends the widely used five ACMG-recommended categories (Fig. 3). To this end, the first step taken was to classify the pathologically relevant variants in the different genes into three functional categories, GoF, LoF and GoP. This allowed us to appropriately perform several cross-gene and cross-variant comparisons, which then enabled us to assign the different genes into two distinct categories in terms of causality; causative genes refer to those genes in which a severe variant can cause CP on its own, whereas disease predisposing genes refer to those genes in which even a highly deleterious variant cannot cause CP by itself. This dichotomy is pivotal because it paves the way for both extension and/or adaptation of the ACMG guidelines (Fig. 3a). Herein, we would like to emphasize that, in common with many term definitions, our currently defined “CP-causing genes” and “CP-predisposing genes” are context-dependent. Thus, we did not consider *CFTR* or *CTRC* as CP-causing genes even if homozygous or compound heterozygous variants in both of them or *CFTR*/*CTRC trans*-heterozygosity might cause CP.

Another key feature of our proposed conceptual framework was the adoption of two thresholds (allele frequency and functional) to differentiate true pathogenic (disease causing) variants from predisposing variants in the context of disease-causing genes, thereby addressing the basic questions raised by Wright et al. [3]. We readily concede that the threshold values we settled upon, particularly the functional ones, may have to be adjusted once more data become available.

Herein, we used CP as a disease model with which to generate a general variant classification framework. This does not mean that a given disorder necessarily always involves both disease causing and disease predisposing genes. Indeed, our analytical approach may well not be applicable across the board to other disease states and in other gene contexts. Rather, it is proposal for a general framework which comprises a five-category classification system for disease-predisposing genes and a seven-category classification system for disease-causing genes, that could potentially be applied to all possible situations. For example, in a truly polygenic disease (i.e., a genetic disorder resulting from the combined action of two or more genes, the implicated genes may in principle be termed disease-predisposing genes and all pathologically relevant variants within these genes may accordingly be classified as “disease-predisposing”. Moreover, in classical autosomal dominant diseases (e.g., autosomal dominant polycystic kidney disease (ADPKD) [70]) or autosomal recessive diseases (e.g., cystic fibrosis [71]), the so-called modifier genes may in principle be termed disease-predisposing genes. Further, the so-called hypomorphic alleles in some disease-causing genes may be classified as “predisposing” (e.g., [72]). Herein, we would like to emphasize that the assignation of a disease gene as disease-causing or disease-predisposing and the establishment of the allele frequency and functional thresholds (in the context of disease-causing genes) would need to be made on a gene-by-gene basis and would require close collaboration between researchers and clinicians with specific expertise in the diseases/genes in question.

It is worth reiterating that this study aimed to provide a proof-of-concept, general variant classification framework, a process facilitated by the availability of functional data for most missense variants in the *PRSS1*, *SPINK1* and *CTRC* genes. It was not however intended to address in detail the specific criteria and rules used to define each variant classificatory category. Therefore, our proposed framework should not be expected to solve all problems of variant interpretation that are likely to be encountered in a clinical exome or genome sequencing context.

It is also worth emphasizing that our proposed general variant classification framework was aimed at classifying variants at individual levels. It was beyond the scope of this study to attempt to classify variants in combination even although such situations are routinely encountered in clinical practice.

The salient point was that it was found to be unnecessary to make more than minimal changes to the five ACMG variant classification categories. As such, all the principles and rules established by ACMG may be readily used and/or adapted for variant classification using our proposed framework.

## Conclusions

In summary, we propose a general classification framework for pathologically relevant variants that successfully addresses key issues pertaining to variant interpretation in medical genetics. The maximal compliance of our proposed five-category and seven-category schemes (for disease-predisposing and disease-causing genes, respectively) with the ACMG guidelines should in principle render these schemes applicable for variant classification in other well-established disease genes.

## Data Availability

All supporting data are available within the article.

## Abbreviations

ACMG-AMP: The American College of Medical Genetics and Genomics and the Association for Molecular Pathology
ACP: Alcoholic chronic pancreatitis
CP: Chronic pancreatitis
ER: Endoplasmic reticulum
FCP: Familial chronic pancreatitis
GoF: Gain-of-function
GoP: Gain-of-proteotoxicity
gpAF: Global population allele frequency
HCP: Hereditary chronic pancreatitis
hspAF: Highest subpopulation allele frequency
ICP: Idiopathic chronic pancreatitis
LoF: Loss-of-function
MAF: Minor allele frequency
*o*/*e*: Observed/expected
OR: Odds ratio
PLoF: Predicted loss-of-function
